# Supplementary material for: Transcriptomic Insights into the Degradation Mechanisms of Fomitopsis pinicola and Its Host Preference for Coniferous over Broadleaf Deadwood
Source: Microorganisms. 2025 Apr 27;13(5):1006. doi: 10.3390/microorganisms13051006 (PMC12113690; doi:10.3390/microorganisms13051006)
Supplement: Supplementary file 1 [file microorganisms-13-01006-s001.zip › microorganisms-3577130-supplementary.pdf]

Supplementary S1

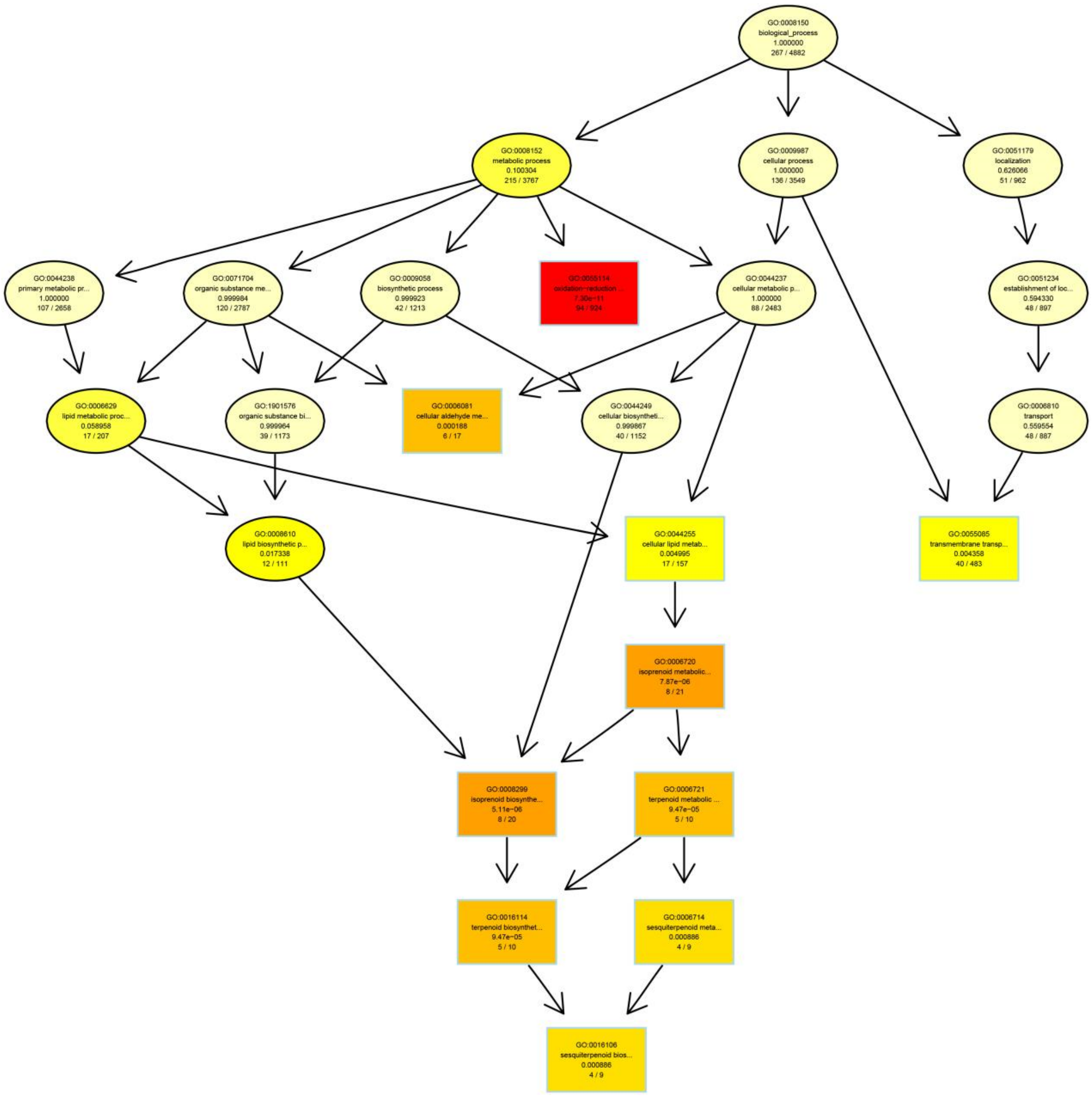

Figure S1. Directed acyclic diagrams of GO terms for FPP vs FPB. A. Directed acyclic diagrams of top10 BP GO terms. Note: Each node represents a GO term, branches represent containment relationships, and the functional scope defined from top to bottom becomes smaller. The top 10 GO terms with the highest significance are set as square, while the other GO terms are set as circular. The darker the color, the more prominent the GO term, and the color ranges from colorless to light yellow to dark yellow to red. The same below.

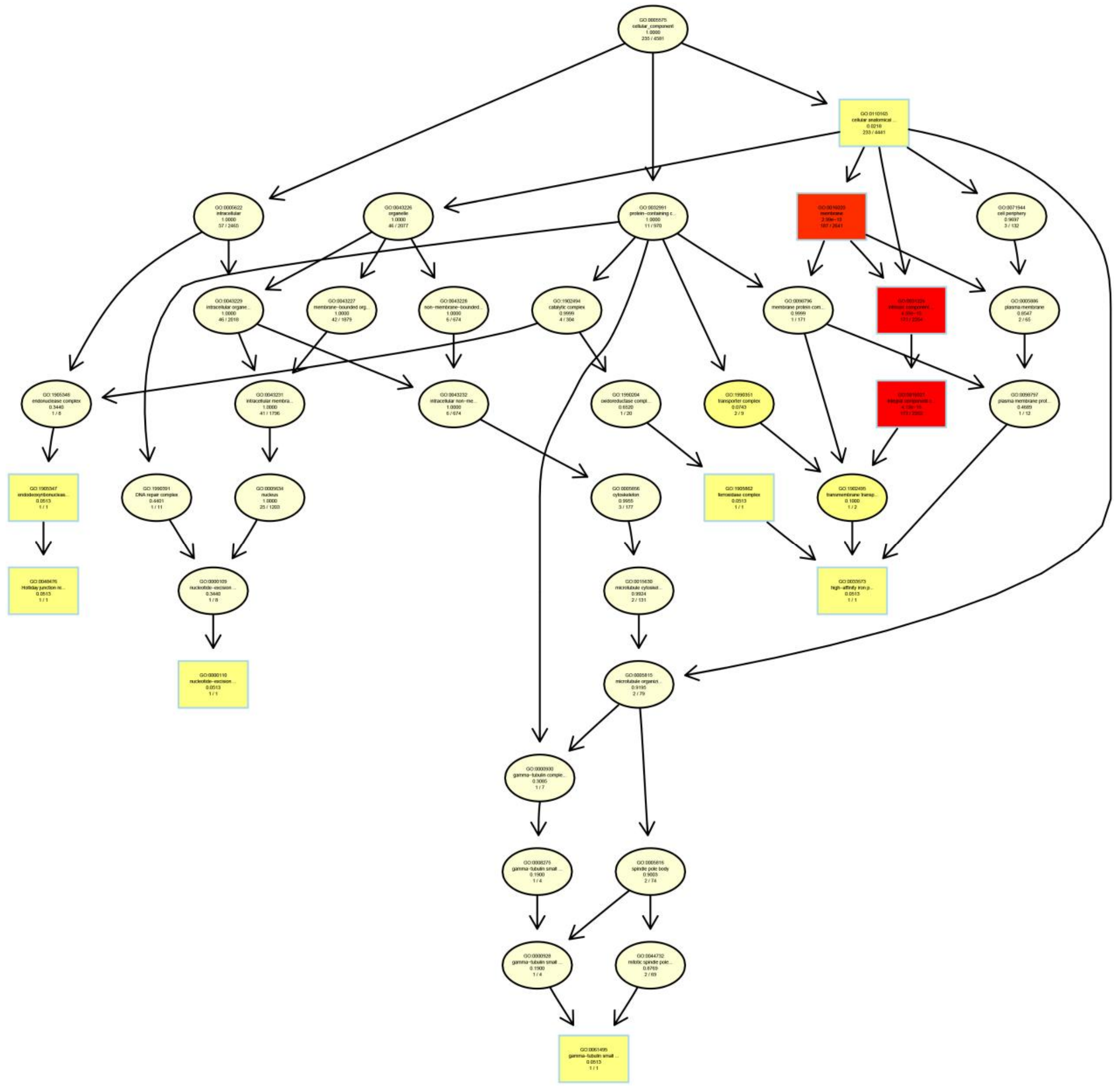

Figure S1. Directed acyclic diagrams of GO terms for FPP vs FPB. B. Directed acyclic diagrams of top10 CC GO terms.

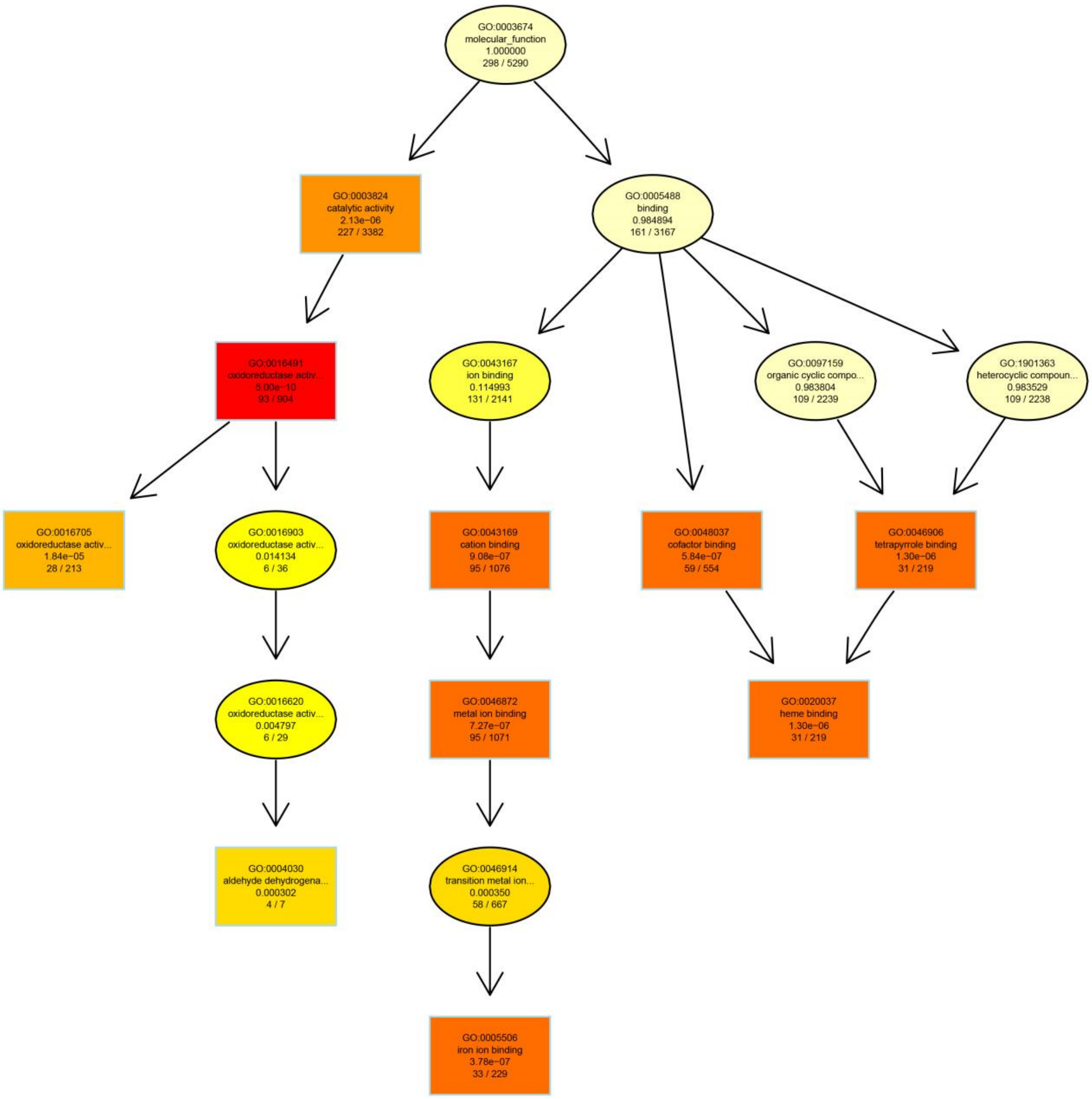

Figure S1. Directed acyclic diagrams of GO terms for FPP vs FPB. C. Directed acyclic diagrams of top10 MF GO terms.

Table S1. GO enrichment and number of DEGs in FPP comparing with FPB (P-value <0.05).

Category	GO.ID	Term	Up	Down	DEG	Total	Pvalue
BP	GO:0055114	oxidation-reduction process	52	42	94	924	7.30268E-11
BP	GO:0008299	isoprenoid biosynthetic process	7	1	8	20	5.11172E-06
BP	GO:0006720	isoprenoid metabolic process	7	1	8	21	7.86955E-06
BP	GO:0006721	terpenoid metabolic process	4	1	5	10	9.46857E-05
BP	GO:0016114	terpenoid biosynthetic process	4	1	5	10	9.46857E-05
BP	GO:0006081	cellular aldehyde metabolic process	4	2	6	17	0.000187689
BP	GO:0006714	sesquiterpenoid metabolic process	3	1	4	9	0.000885837
BP	GO:0016106	sesquiterpenoid biosynthetic process	3	1	4	9	0.000885837
BP	GO:0055085	transmembrane transport	15	25	40	483	0.004357987
BP	GO:0044255	cellular lipid metabolic process	16	1	17	157	0.00499511
BP	GO:0098869	cellular oxidant detoxification	4	1	5	26	0.012018169
BP	GO:0009082	branched-chain amino acid biosynthetic process	0	4	4	18	0.014561877
BP	GO:0098754	detoxification	4	1	5	28	0.016427619
BP	GO:1990748	cellular detoxification	4	1	5	28	0.016427619
BP	GO:0008610	lipid biosynthetic process	11	1	12	111	0.017338389
BP	GO:0097237	cellular response to toxic substance	4	1	5	31	0.024866026
BP	GO:0009081	branched-chain amino acid metabolic process	0	4	4	21	0.025046312
BP	GO:1901617	organic hydroxy compound biosynthetic process	4	0	4	21	0.025046312
BP	GO:0006513	protein monoubiquitination	1	1	2	5	0.026697285
BP	GO:0006821	chloride transport	0	2	2	5	0.026697285
BP	GO:0015976	carbon utilization	2	0	2	5	0.026697285
BP	GO:0006637	acyl-CoA metabolic process	3	0	3	13	0.030747892
BP	GO:0035383	thioester metabolic process	3	0	3	13	0.030747892
BP	GO:0046165	alcohol biosynthetic process	3	0	3	13	0.030747892
BP	GO:0009636	response to toxic substance	4	1	5	33	0.03179956
CC	GO:0016021	integral component of membrane	120	53	173	2252	4.12166E-15
CC	GO:0031224	intrinsic component of membrane	120	53	173	2254	4.58551E-15
CC	GO:0016020	membrane	131	56	187	2641	2.98861E-13
CC	GO:0110165	cellular anatomical entity	157	76	233	4441	0.021813802
MF	GO:0016491	oxidoreductase activity	53	40	93	904	5.00079E-10
MF	GO:0005506	iron ion binding	22	11	33	229	3.77853E-07
MF	GO:0048037	cofactor binding	36	23	59	554	5.84157E-07
MF	GO:0046872	metal ion binding	62	33	95	1071	7.26615E-07
MF	GO:0043169	cation binding	62	33	95	1076	9.07898E-07
MF	GO:0020037	heme binding	20	11	31	219	1.30165E-06
MF	GO:0046906	tetrapyrrole binding	20	11	31	219	1.30165E-06
MF	GO:0003824	catalytic activity	141	86	227	3382	2.13455E-06

MF	GO:0016705	oxidoreductase activity, acting on paired donors, with incorporation or reduction of molecular oxygen	20	8	28	213	1.84287E-05
MF	GO:0004030	aldehyde dehydrogenase [NAD(P)+] activity	3	1	4	7	0.00030174
MF	GO:0046914	transition metal ion binding	37	21	58	667	0.000349676
MF	GO:0004497	monooxygenase activity	16	6	22	179	0.00039676
MF	GO:0004190	aspartic-type endopeptidase activity	10	1	11	63	0.000667008
MF	GO:0070001	aspartic-type peptidase activity	10	1	11	63	0.000667008
MF	GO:0010334	sesquiterpene synthase activity	3	1	4	9	0.000992332
MF	GO:0045482	trichodiene synthase activity	3	1	4	9	0.000992332
MF	GO:0004175	endopeptidase activity	16	2	18	144	0.001092339
MF	GO:0046912	transferase activity, transferring acyl groups, acyl groups converted into alkyl on transfer	1	3	4	11	0.002375056
MF	GO:0016829	lyase activity	9	5	14	114	0.004505064
MF	GO:0016620	oxidoreductase activity, acting on the aldehyde or oxo group of donors, NAD or NADP as acceptor	4	2	6	29	0.00479708
MF	GO:0016835	carbon-oxygen lyase activity	6	2	8	48	0.004834803
MF	GO:0004348	glucosylceramidase activity	3	0	3	7	0.005227165
MF	GO:0010333	terpene synthase activity	3	1	4	14	0.006297884
MF	GO:0008233	peptidase activity	21	3	24	252	0.007399275
MF	GO:0050662	coenzyme binding	14	13	27	297	0.008531499
MF	GO:0070011	peptidase activity, acting on L-amino acid peptides	19	3	22	229	0.009209791
MF	GO:0016209	antioxidant activity	4	1	5	26	0.013567312
MF	GO:0016903	oxidoreductase activity, acting on the aldehyde or oxo group of donors	4	2	6	36	0.014133847
MF	GO:0016838	carbon-oxygen lyase activity, acting on phosphates	3	1	4	18	0.016113011
MF	GO:0008236	serine-type peptidase activity	8	1	9	77	0.027981875
MF	GO:0016825	hydrolase activity, acting on acid phosphorus-nitrogen bonds	8	1	9	77	0.027981875
MF	GO:0017171	serine hydrolase activity	8	1	9	77	0.027981875
MF	GO:0008198	ferrous iron binding	1	1	2	5	0.028237837
MF	GO:0004553	hydrolase activity, hydrolyzing O-glycosyl compounds	8	7	15	160	0.034954316
MF	GO:0016798	hydrolase activity, acting on glycosyl bonds	9	7	16	176	0.038352345
MF	GO:0004252	serine-type endopeptidase activity	6	0	6	45	0.038810167
MF	GO:0010181	FMN binding	2	4	6	45	0.038810167

MF	GO:0003700	DNA-binding transcription factor activity	9	2	11	111	0.046926981
